# Visualisation of cholesterol and ganglioside GM1 in zebrafish models of Niemann–Pick type C disease and Smith–Lemli–Opitz syndrome using light sheet microscopy

**DOI:** 10.1007/s00418-020-01925-2

**Published:** 2020-10-20

**Authors:** Sophie R. Cook, Cerys Bladen, Johanna Smith, Emily Maguire, Jordan Copner, Gareth D. Fenn, Kim Wager, Helen Waller-Evans, Emyr Lloyd-Evans

**Affiliations:** 1 School of Biosciences, Cardiff University, Sir Martin Evans Building, Museum Avenue, Cardiff, CF10 3AX UK; 2 Medicines Discovery Institute, Cardiff University, Main Building, Park Place, Cardiff, CF10 3AT UK; 3 Present Address: Oxford Pharmagenesis, Tubney Warren Barn, Tubney, Oxford, OX13 5QJ UK

**Keywords:** Light sheet microscopy, Cholesterol, Ganglioside GM1, Niemann–Pick type C, Smith–Lemli–Opitz syndrome

## Abstract

Lysosomal storage diseases are the most common cause of neurodegeneration in children. They are characterised at the cellular level by the accumulation of storage material within lysosomes. There are very limited therapeutic options, and the search for novel therapies has been hampered as few good small animal models are available. Here, we describe the use of light sheet microscopy to assess lipid storage in drug and morpholino induced zebrafish models of two diseases of cholesterol homeostasis with lysosomal dysfunction: First, Niemann–Pick type C disease (NPC), caused by mutations in the lysosomal transmembrane protein NPC1, characterised by intralysosomal accumulation of cholesterol and several other lipids. Second, Smith–Lemli–Opitz syndrome (SLOS), caused by mutations in 7-dehydrocholesterol reductase, which catalyses the last step of cholesterol biosynthesis and is characterised by intralysosomal accumulation of dietary cholesterol. This is the first description of a zebrafish SLOS model. We find that zebrafish accurately model lysosomal storage and disease-specific phenotypes in both diseases. Increased cholesterol and ganglioside GM1 were observed in sections taken from NPC model fish, and decreased cholesterol in SLOS model fish, but these are of limited value as resolution is poor, and accurate anatomical comparisons difficult. Using light sheet microscopy, we were able to observe lipid changes in much greater detail and identified an unexpected accumulation of ganglioside GM1 in SLOS model fish. Our data demonstrate, for the first time in zebrafish, the immense potential that light sheet microscopy has in aiding the resolution of studies involving lysosomal and lipid disorders.

**Electronic supplementary material:**

The online version of this article (10.1007/s00418-020-01925-2) contains supplementary material, which is available to authorized users.

## Introduction

Lysosomal storage diseases (LSDs) are a group of ~ 70 inherited metabolic disorders, nearly all of which are characterised by intra-lysosomal accumulation of storage material, neurodegeneration and peripheral involvement (Cox and Cachón-González 2012). Together, LSDs are the most common cause of childhood neurodegeneration often causing early mortality, but at present with very few therapeutic options (Onyenwoke and Brenman 2015). It is, therefore, important to understand disease aetiology, especially in vivo, to discover new therapeutic targets and develop new treatments for these diseases.

Animal models of LSDs are available across a range of species including mouse, rat, dog, cat, pig and zebrafish (Bond et al. 2013; Xu et al. 2016). Mice are most commonly used, but are often poor models of disease progression and hallmarks, reviewed here (Favret et al. 2020). Increasingly, large mammals are used to model LSDs; these models offer advantages in translation of effects to humans, particularly due to their larger more complex brain (Gurda and Vite 2019). However, these experiments are both slow and costly (Zhang and Peterson 2020).

Zebrafish (*Danio rerio*) LSD models have risen dramatically over the past 20 years, now with more than 20 different LSDs, reviewed here (Zhang and Peterson 2020). Zebrafish are extremely useful for therapeutic advancement as they provide a model that is cheap, quick to breed and fast to develop. Zebrafish lay eggs that are fertilised externally and at predictable times of day; they are also particularly amenable to genetic manipulation, e.g. by morpholino, CRISPR-Cas9 and TALENs (Adamson et al. 2018). Zebrafish share a high degree of genetic similarity to humans, with zebrafish homologues for 71% of human genes (Howe et al. 2013). This is especially evident with regard to disease modelling, as 82% of human disease-associated genes have zebrafish homologues (Howe et al. 2013). A further growing advantage of zebrafish is their utility in high-throughput drug screening and toxicology assays, work that can significantly accelerate and reduce the costs of drug discovery (Wiley et al. 2017). Finally, a major advantage over larger animals is their optical transparency in the larval stages, allowing easy visualisation of stains in vivo, without the need for sectioning (Zhang and Peterson 2020). One possible drawback of zebrafish is the lack of effective and accurate antibodies, especially for lysosomal proteins.

A key LSD characteristic, alongside expansion of the lysosomal system, is the intra-lysosomal accumulation of storage materials, primarily lipids. Cholesterol and glycosphingolipids, gangliosides GM1 and GM2, form the primary storage material in the LSDs Niemann–Pick type C (NPC) and GM1/GM2 gangliosidosis, respectively, but have also been found as secondary storage material in many other LSDs (Lloyd-Evans and Platt 2010; Walkley and Vanier 2009). Considering the widespread presence of cholesterol and gangliosides as secondary storage in LSDs, it is fortunate that well-characterised probes to image these lipids exist (Viljetić et al. 2012; Chisada et al. 2009). These include filipin, a blue fluorescent polyene antibiotic that selectively binds cholesterol (Schroeder et al. 1971; Kruth and Vaughan 1980), and cholera toxin subunit B (CtxB), produced by *Vibrio cholerae* bacteria and that selectively binds ganglioside GM1, which is present in zebrafish, as is ganglioside GM2 (Holmgren et al. 1973; Saslowsky et al. 2010; Boutry et al. 2018). These probes are specific and have been used extensively in cellular and tissue section studies of LSDs, including secondary storage (Arthur et al. 2011; Walkley et al. 1998) across species (Pagano 2003; Blanchette-Mackie et al. 1989).

This study focuses on imaging lipids in zebrafish models of two diseases of sterol homeostasis: NPC and Smith–Lemli–Opitz syndrome (SLOS). NPC is a multi-lipid storage disorder, characterised by intra-lysosomal accumulation of cholesterol, gangliosides and multiple other lipids in most tissues (Lloyd-Evans et al. 2008). It is caused by loss of function mutations in the *NPC1* gene, and patients present with a progressive neurodegenerative disease that includes ataxia and hallmarks of dementia (Lloyd-Evans and Platt 2010).

Unlike NPC, which is a sterol transport disease, SLOS is a sterol biosynthesis disease, caused by loss of function mutations in the *DHCR7* gene (Waterham et al. 1998). SLOS is a developmental disorder; symptoms include birth defects such as microcephaly, cleft palate and limb anomalies (Kelley and Hennekam 2000). The *DHCR7* gene encodes an ER membrane protein, 3β-hydroxysterol Δ^7^-reductase (DHCR7), that catalyses the last step of cholesterol biosynthesis, converting 7-dehydrocholesterol (7-DHC) to cholesterol. This leads to reduced endogenous cholesterol production and accumulation of 7-DHC in cells and tissues which is thought to underlie the developmental defects via alterations to Sonic hedgehog signalling (Moebius et al. 1998). A therapeutic approach to increase dietary cholesterol has been shown to be ineffective (Tierney et al. 2010), which may be due to the NPC-like lysosomal low-density lipoprotein (LDL)-derived cholesterol storage reported in SLOS patient cells (Wassif et al. 2002; Platt et al. 2014). This would prevent the exit of dietary cholesterol from lysosomes and its utilisation by the biosynthetic enzymes in the ER. Animal models of SLOS are poor; a drug-induced rat model using the DHCR7 inhibitor, AY9944, being amongst the better current options (DeBarber et al. 2011). Zebrafish may provide a new tool to study this disease and to further study in situ the extent of the endo-lysosomal LDL-cholesterol transport defect, which could improve therapeutic options for SLOS.

In this paper we highlight the use of light sheet microscopy as a more robust and effective method to visualise toxin- and fluorescence-based lipid markers for studying lipid storage in zebrafish larvae. Owing to the dearth of good antibodies available for zebrafish lysosomal proteins, we believe that this combination of light sheet imaging with lipid markers could widen the use and characterisation of zebrafish models of LSDs.

## Materials and methods

Unless otherwise stated, all reagents were purchased from Sigma-Aldrich or ThermoFisher.

### Zebrafish maintenance

#### Animal welfare statement

This study only used wild-type adults for breeding; all larvae were used prior to 5 days post fertilization and were euthanised using tricaine at the end of all experiments. All animal experiments were carried out in accordance with EU directive 2010/63/EU and the UK Animal Welfare Act, 2006.

#### Husbandry

Wild-type AB/TL zebrafish, sourced from the Division of Biosciences, University College London, were housed in a multi-rack aquarium system in the School of Biosciences aquarium, Cardiff University at 28 °C on a 14 h light, 10 h dark cycle. To obtain embryos, pairs of fish were placed in breeding tanks overnight, and eggs collected. Embryos were separated into experimental groups and maintained at 28 °C in E3 medium (5 mM NaCl, 0.17 mM KCl, 0.33 mM CaCl_2_, 0.33 mM MgSO_4_, 0.1% methylene blue) in 24-well plates.

#### Drug treatments

Embryos were incubated in drug treatments (2 μg/ml U18666A, 50 or 75 μM AY9944, 75 μM trazodone, 500 μM miglustat (Toronto Research Chemicals, North York, Canada)), separately or in combination, from 6 h post-fertilisation (hpf), with drug treatments refreshed daily. Control fish were raised in E3 medium containing an equivalent percentage of sterile anhydrous DMSO, which in no case exceeded 0.25%. Drug treatments were diluted from concentrated DMSO (U18666A, AY9944 and trazodone) or water (miglustat) stocks in E3 medium, with no more than 0.25% total DMSO. Dead fish (determined by disintegration or lack of heartbeat) were removed daily. No significant toxicity was observed in any of the drug treatments.

To generate *npc1* and *dhcr7* morphant embryos, 3.5 ng morpholino (MO) anti-sense oligonucleotides targeting the start ATG of *npc1*, *dhcr7* and *p53* messenger RNA (Gene Tools, Philomath, USA), diluted in Danieau’s solution (58 mM NaCl, 0.7 mM KCl, 0.4 mM MgSO_4_, 0.6 mM Ca(NO_3_)_2_, 5 mM HEPES, pH 7.6) with 1% phenol red, were pressure-injected using a Narishige UM-1PF micromanipulator (Narishige Group Global, London, UK) and Warner PLI-10 pico-liter injector (Warner Instruments, Hamden, USA) into one- or two-cell stage embryos using a glass capillary injection needle. MO sequences: *npc1*—5′-TGTGGTTTCTCCCCAGCAGAAGCAT-3′ (Schwend et al. 2011), *dhcr7*—5′-TCACCCTGTCAGATGCCATCATG-3′, *p53*—5′-GCGCCATTGCTTTGCAAGAATTG-3’.

### Light microscopy

Images were taken using an AmScope stereo dissection microscope (AmScope, Irvine, USA) with Watec WAT-902H monochrome camera (Watec, Yamagata-Ken, Japan) and Corel Video Studio 11 image capture software (Corel, Ottawa, Canada), and brightness and contrast adjusted using Adobe Photoshop CS6 (Adobe, San Jose, USA).

### Behavioural analyses

Spontaneous coiling and escape response analyses were carried out, with minor modifications, as described in Wager et al. (2016)*.* Briefly:

#### Spontaneous coiling

Groups of 20 embryonic zebrafish at 24 hpf were arrayed in a petri dish containing E3 medium and placed on an AmScope stereo dissection microscope. Three-minute recordings were taken at 30 frames per second using a Watec WAT-902H monochrome camera and Corel Video Studio 11 image capture software, and coiling events counted manually for each embryo.

#### Escape response

Individual 72 hpf larvae were placed, in a petri dish containing E3 medium, on an AmScope stereo dissection microscope, with the larva in the centre of the field of view. Fish were lightly touched on the tail with a pipette tip until an escape response was elicited. Recordings were taken at 30 frames per second using a Watec WAT-902H monochrome camera and Corel Video Studio 11 image capture software. Movies were watched in slow motion, and the time (in s) for the fish to leave the field of view recorded.

#### Heart rate

Individual 72 hpf larvae were placed, in a petri dish containing E3 medium, on an AmScope stereo dissection microscope, with the larva in the centre of the field of view. One-minute recordings were made at 240 frames per second using a GoPro Hero 3 camcorder (GoPro, San Mateo, USA). Movies were watched in slow motion, and the number of heart beats per minute recorded.

### Lysotracker green staining

To visualize lysosomes, live 72 hpf zebrafish were stained with lysotracker green as described in Wager et al. (2016). Briefly, larvae were incubated in 10 μM lysotracker green in phosphate-buffered saline (PBS) for 30 min at room temperature in the dark with agitation and washed three times for 5 min in PBS. Larvae were lightly anaesthetised with tricaine, and images taken with a Zeiss LSM 880 upright confocal microscope with Airyscan super resolution detector using the Zen 2 LSM software package (Zeiss, Oberkochen, Germany).

### Lipid visualization by light sheet microscopy

#### Filipin staining

Larvae were fixed in 4% paraformaldehyde in PBS, overnight at 4 °C, and washed three times for 10 min in PBS containing 0.1% Triton X-100 (PBST). Larvae were blocked for 1 h in blocking buffer (PBST containing 5% goat serum and 1% BSA), and incubated in 187.5 μg/ml filipin, diluted in blocking buffer, for 4 h in the dark at room temperature with agitation. Larvae were washed a further three times for 10 min in PBST, stored at 4 °C in the dark and imaged within 1–2 weeks.

#### CtxB staining

Larvae were fixed in 4% paraformaldehyde in PBS overnight at 4 °C and washed three times for 10 min in PBST. Larvae were incubated in blocking buffer for 1 h at room temperature and incubated overnight in 2.5 μg/ml FITC-CtxB, diluted in blocking buffer, overnight at 4 °C in the dark with agitation. Following three 10-min washes in PBST, stained larvae were stored at 4 °C and imaged within 1–2 weeks.

#### Light sheet imaging

Stained larvae were embedded in low melting point agarose and imaged using a Zeiss Light sheet Z.1 single plane illumination microscope, with a single sCMOS PCO.Edge camera and Zen Black software. Filipin was imaged using the 405 nm laser and 430 nm emission filter, CtxB using the 470 nm laser and 520 nm emission filter. Laser power was kept as low as possible to avoid bleaching and never exceeded 25%. Maximum intensity projection images were exported and brightness and contrast adjusted equally for all conditions using Adobe Photoshop CS6.

### Statistical analysis

All behavioural data were analysed by one-way ANOVA with Tukey’s post-hoc correction (normally distributed) or Kruskal–Wallis test with Dunn’s post-hoc correction (not normally distributed) following determination of normal distribution by D’Agostino and Pearson omnibus normality test.

### Supplemental methods

#### Lipid visualization in cryosections

#### Cryosectioning

Drug or MO treated 96 hpf larvae were fixed overnight in 4% paraformaldehyde in PBS at 4 °C. Larvae were washed three times in PBS, cryoprotected overnight in PBS containing 30% sucrose at 4 °C, immersed in TissueTek OCT (Sakura Finetek, Tokyo, Japan) and frozen slowly by lowering into isopentane cooled with dry ice. Thin (12 μm) sections were taken using a Bright 5000 cryostat (Bright Instruments, Luton, UK) onto X-tra adhesive slides (Leica Biosystems, Milton Keynes, UK), which were air dried and stored at − 80 °C until stained.

#### Filipin staining (cholesterol)

Sections were allowed to defrost to room temperature, washed three times for 5 min in PBS containing 0.1% Triton X-100 (PBST), and incubated in 187.5 μg/ml filipin, diluted in PBST containing 5% foetal bovine serum (FBS) and 1% bovine serum albumin (BSA), for 45 min in the dark at room temperature. Sections were washed three times for 5 min in PBST, coverslipped using fluoroshield, and stored in the dark until imaged.

#### Cholera toxin subunit B (CtxB) staining (ganglioside GM1)

Sections were allowed to defrost to room temperature, washed three times for 5 min in PBST, incubated in blocking solution (PBST containing 1% BSA) for 30 min at room temperature, and incubated in 2.5 μg/ml FITC-CtxB, diluted in blocking solution, overnight at 4 °C in the dark. Sections were washed three times for 5 min in PBST, coverslipped using fluoroshield and stored in the dark until imaged.

#### Imaging

Sections were imaged using a Zeiss Colibri LED fluorescence microscope, Axiocam Mrm monochrome charged coupled device camera and Axiovision 4.8 software. Excitation and emission wavelengths were as follows: filipin—380 nm excitation, 430 nm emission, FITC-CtxB—470 nm excitation, 520 nm emission. Brightness and contrast were adjusted equally for all conditions using Adobe Photoshop CS6.

### Lipid dot-blots

Lipid standards (cholesterol, sphingomyelin, ceramide, sphingosine, phosphatidylserine, glucosylceramide, lyso-(bis)phosphatidic acid, mixed gangliosides (Sigma-Aldrich; Larodan, Solna, Sweden; or Avanti Polar Lipids, Alabaster, USA)) were dissolved in ethanol or a 1:1 chloroform:methanol mix at 2 mg/ml. Lipids (2 μl) were spotted onto Immobilon-P PVDF membranes and allowed to dry. Membranes were incubated in blocking buffer (PBS containing 5% goat serum and 1% BSA) for 1 h at room temperature, followed by incubation in 187.5 μg/ml filipin, diluted in blocking buffer, for 4 h at room temperature, or in 0.5 μg/ml FITC-CtxB, diluted in blocking buffer, overnight at 4 °C. All incubations took place in the dark with agitation. Following three 10-min washes in PBS, membranes were viewed using a Biorad EZ gel documentation system (Biorad, Hercules, USA) using the UV (filipin) and blue (FITC-CtxB) settings. Brightness and contrast were adjusted using Adobe Photoshop CS6.

### Thin layer chromatography

Total lipids were extracted from 96 hpf wild-type zebrafish larvae in 1:2 (v/v) chloroform:methanol, as described (te Vruchte et al. 2004). Samples were centrifuged for 5 min at 1000 rpm to remove sediment and washed three times by phase separation. For each wash, 1 ml chloroform and 1 ml PBS were added, samples centrifuged for 5 min at 1000 rpm, and the aqueous (upper) phase removed. The final organic phase was dried under N_2_ and purified lipids resuspended in ethanol. Lipids were separated on high-performance thin layer silica gel 60 chromatography (HPTLC) plates using a 65:25:4 chloroform:methanol:H_2_O solvent system and visualised using p-anisaldehyde as previously described (Maue et al. 2012). Lipid standards, from a total porcine brain extract, were from Avanti Polar Lipids.

## Results and discussion

### NPC disease zebrafish model phenotypes

**Fig. 1:**
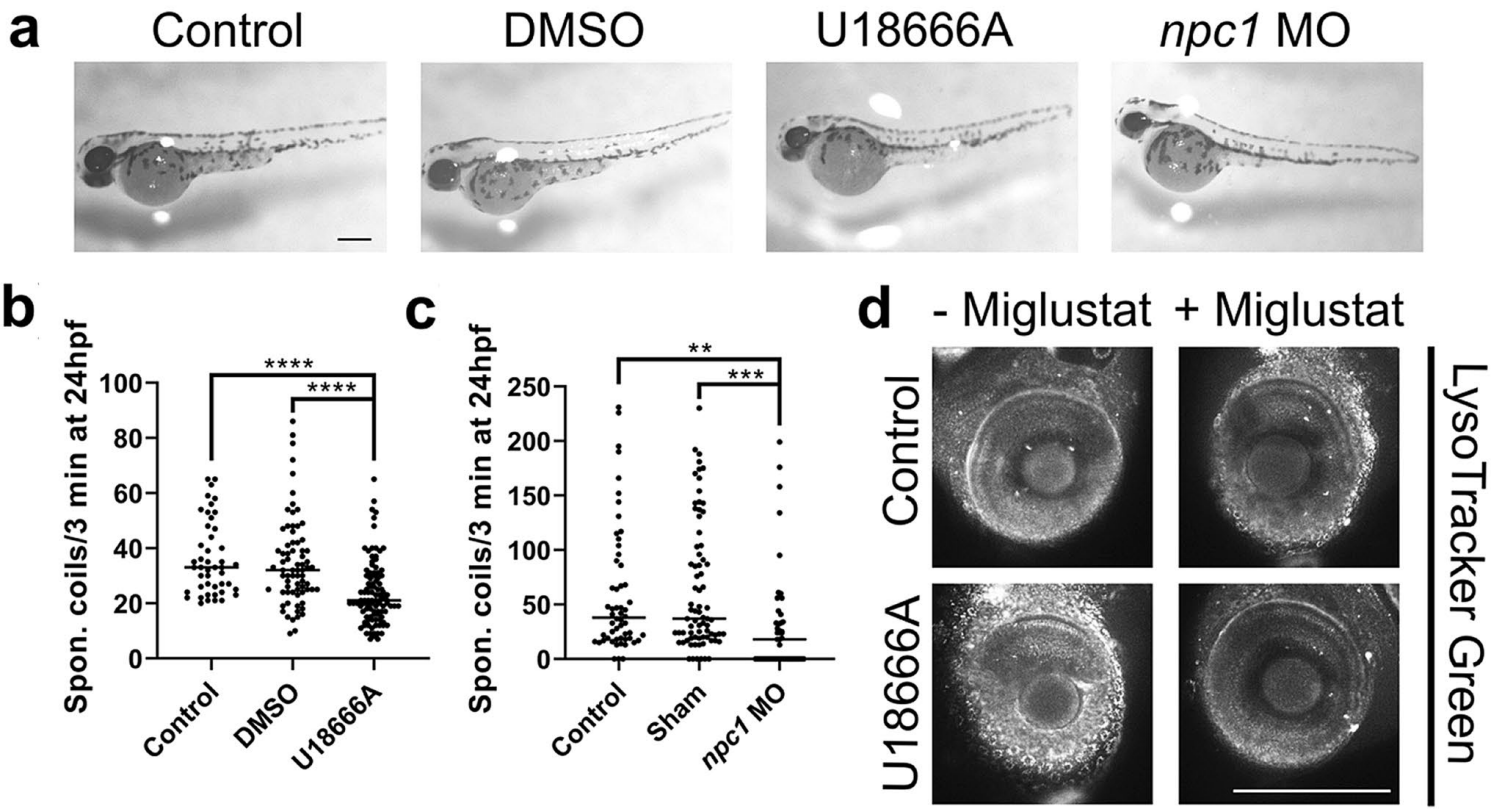
Identification of NPC phenotypes in a Npc1 inhibitor and *npc1* morpholino zebrafish larvae model. NPC phenotypes were induced in the zebrafish larvae using either the Npc1 inhibitor, U18666A (2 µg/ml), or *npc1* morpholino (*npc1* MO) injected at the 1–2 cell stage with DMSO and Sham controls as necessary; **a** Morphology of 48 h post fertilisation (hpf) zebrafish by light microscopy. Quantitative behavioural analysis of 24 hpf zebrafish measuring number of spontaneous coils over 3 min in 2 µg/ml U18666A treated **(b)** and *npc1* morphant **(c)** zebrafish, *p < 0.05, **p < 0.01, ****p < 0.0001, Kruskal–Wallis test with Dunn’s post-hoc correction. Representative images of LysoTracker Green staining of 96 hpf control and U18666A treated zebrafish larvae eyes with and without 500 µM miglustat co-treatment **(d)**. Scale bars = 280 µm. *N* = 3–5, with a minimum of 10 fish per *N*

Two separate methods were utilized to induce NPC in zebrafish larvae: a known mammalian Npc1 inhibitor, U18666A (Lu et al. 2015), or *npc1* morpholino. We compared both drug and morpholino models to confirm that the morpholino phenotypes are not caused by off-target effects. Expression of Npc1 in zebrafish has been demonstrated by Tseng et al (2018). Treatment with 2 µg/ml U18666A did not cause any gross morphological defects at 48 hpf (Fig. 1a), indicating there were no major off-target effects impacting on larvae morphology or development.

NPC is characterised by progressive motor defects (Vanier et al. 1988), so it was not unexpected to observe behavioural changes in both the 2 µg/ml U18666A treated and *npc1* morphant larvae at 24 hpf (Fig. 1b, c). Rate of spontaneous coiling is a measure of neuromuscular development at 1–2 days post fertilisation. Treatment with 2 μg/ml U18666A caused an ~ 1.5-fold reduction in spontaneous coiling over 3 min at 24 hpf. This phenotype was confirmed in the *npc1* morphant, which also caused a spontaneous coiling defect at 24 hpf, with a similar ~ 1.9-fold reduction.

Having confirmed the presence of a disease-specific phenotype, we next determined whether lysosomal storage was present at 96 hpf using the live lysosomal marker Lysotracker Green. Super resolution confocal microscopy staining of lysosomal volume using Lysotracker Green showed a fluorescence increase in the eye of live (sedated) 2 µg/ml U18666A treated fish compared to controls (Fig. 1d), indicating the presence of lysosomal storage, also reported in Npc1 null zebrafish larvae (Tseng et al. 2018). The eye was chosen as this was the only area where clear images of lysosomal storage could be generated by confocal microscopy. We confirmed that this increase represented lysosomal storage, and not U18666A autofluorescence, by coincubation of larvae with both U18666A and the substrate reduction therapy, miglustat. Miglustat (*N*-butyldeoxynojirimycin) is an approved NPC therapy that inhibits glycosphingolipid biosynthesis (including gangliosides) and reduces lysosomal storage in NPC (Zervas et al. 2001b; te Vruchte et al. 2004). Co-treatment with 500 μM miglustat for 4 days normalised the level of Lysotracker Green staining (Fig. 1d). This confirms that the lysosomal expansion is due to U18666A inhibition of Npc1*,* and likely lysosomal lipid storage*,* rather than off-target effects, as have been suggested (Wassif et al. 2002; Bae and Paik 1997).

After confirming lysosomal storage, we next identified storage products. We did not confirm that this storage is lysosomal by co-staining with lysotracker, as lysotracker fluorescence is thought to be dependent on protonation, and staining is not maintained, or is only present in large organelles, following fixation, even with Lysotracker Red DND-99, marketed as aldehyde fixable, making it unsuitable for co-staining in fixed tissue (ThermoFisher). Additionally, the focus of this study was on the overall lipid burden in these diseases, rather than the subcellular localisation. Cholesterol is considered by many as the primary storage material in NPC, despite indications otherwise (Malathi et al. 2004; Lloyd-Evans and Platt 2010; Feldman et al. 2015) and is, therefore, an important phenotype to measure in the NPC zebrafish models. Using fixed sections (72 hpf), increased levels of filipin staining were observed throughout the brain of 2 µg/ml U18666A-treated or *npc1* morphant fish compared to controls (Supplemental Figure 1a). The specificity of filipin for cholesterol is shown in Supplemental Figure 2a. We also used CtxB to detect ganglioside GM1 (specificity shown in Supplemental Figure 2b) in sections of 72 hpf larvae. Increased levels of CtxB staining were observed throughout the brain of *npc1* morphant fish compared to controls (Supplemental Figure 1b), indicative of increased ganglioside GM1 deposition. This mirrors previous findings that ganglioside GM1 accumulates as secondary storage material in NPC (Lloyd-Evans et al. 2008) and confirms ganglioside GM1 storage as a phenotype in the *npc1* morpholino model. We, and others, have confirmed the presence of gangliosides in zebrafish larvae by HPTLC (Supplemental Figure 2c) (Saslowsky et al. 2010; Viljetić et al. 2012; Chisada et al. 2009). The presence of substantial cholesterol and ganglioside GM1 storage (Fig. 4b and Supplemental Figure 1a and b) in the NPC model zebrafish also validates these models, as these lipids do not accumulate substantially together in any other disease.

### SLOS zebrafish model phenotypes

Smith–Lemli–Opitz disease (SLOS) phenotypes were induced in zebrafish larvae using either the small molecule inhibitors of mammalian DHCR7, AY9944 or trazodone (DeBarber et al. 2011; Hall et al. 2013), or *dhcr7* morpholino. We did not observe any p53 mediated toxicity in *dhcr7* morphant larvae, and co-injection with *p53* morpholino did not alleviate the *dhcr7* morpholino-induced phenotypes. We have, therefore, used *dhcr7* morpholino alone, with wild-type and sham injected controls. No *p53* morpholino control was deemed necessary for Npc1 as no p53 mediated toxicity was observed in earlier studies using the same morpholino (Schwend et al. 2011).

**Fig. 2:**
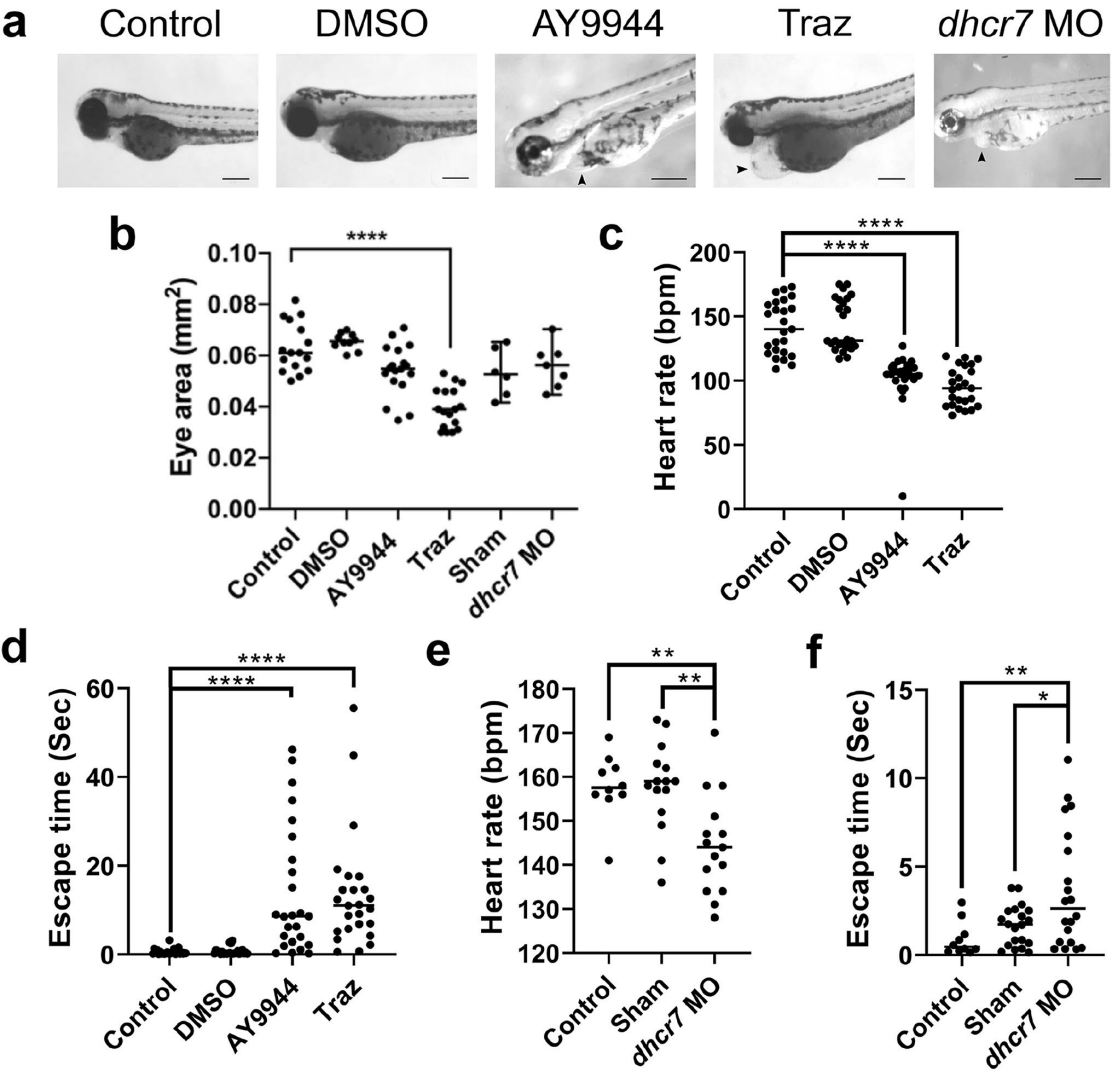
Identification of SLOS phenotypes in Dhcr7 inhibitor and *dhcr7* morpholino zebrafish larvae models. SLOS phenotypes were induced in the zebrafish larvae using either Dhcr7 inhibitors AY9944 (75 µM) or trazodone (traz, 75 µM), or *dhcr7* morpholino (*dhcr7* MO) injected at the 1–2 cell stage with DMSO and Sham controls as necessary; **a** Morphology of 72 hpf zebrafish by light microscopy. Arrowheads indicate heart defects. Quantitative analysis of 72 hpf zebrafish by light microscopy; **b** eye area. Quantitative behavioural analysis of 72 hpf inhibitor treated zebrafish; **c** heart rate over 1 min and **d** escape response time. Quantitative behavioural analysis of 72 hpf *dhcr7* morphant zebrafish; **e** heart rate over one minute and **f** escape response time. All analysis by Kruskal–Wallis test with Dunn’s post-hoc correction (**b**, **c**, **d**) or one-way ANOVA with Tukey post-hoc correction (**e**, **f**). *p < 0.05, **p < 0.01, ****p < 0.0001. Scale bars = 280 µm. *N* = 3, with a minimum of 3–10 fish per *N*

Neither 75 µM AY9944 nor 75 µM trazodone caused pigmentation changes at 72 hpf. However, 75 µM trazodone treatment caused a clear distention of the pericardial sac, also present, albeit to a much lesser extent, in 75 µM AY9944 treated and *dhcr7* morphant fish (Fig. 2a). The eye area of fish treated with 75 µM trazodone is reduced ~ 1.8-fold compared to controls, while neither 75 µM AY9944 treatment nor *dhcr7* morpholino had any effect on eye area (Fig. 2b). As this phenotype is not replicated by AY9944 treatment, and eye/vision defects are not reported in SLOS patients, this change in eye area is unlikely to be SLOS specific but is more likely an off-target effect of trazodone. This is not surprising, as trazodone is known to inhibit multiple proteins (Kraus et al. 2007) and causes visual issues and an increased risk of eye-related conditions as side effects when used as an antidepressant (Pae et al. 2003; Halikas 1995).

As morphological defects were observed in the pericardial sac at 72 hpf, heart rate was investigated. Treatment with both 75 µM AY9944 and 75 µM trazodone reduced heart rate at 72 hpf (Fig. 2c), by ~ 1.4 and 1.5-fold, respectively, compared to control. Congenital heart defects are reported in about 50% of SLOS patients, supporting the detection of heart rate and pericardial sac defects in the model fish as SLOS phenotypes (Lin et al. 1997; Digilio et al. 2003). As SLOS, like NPC, is characterised by progressive motor defects we were not surprised to identify behavioural phenotypes in the fish. Escape response time was significantly increased in both 75 µM trazodone and 75 µM AY9944 treatments at 72 hpf with respective ~ 24 and 23-fold increases compared to control (Fig. 2d). Both the heart rate and escape response defects were confirmed as SLOS specific phenotypes, rather than off-target effects of the drugs, using *dhcr7* morpholino. The *dhcr7* morpholino caused a significant ~ 1.1-fold decrease in heart rate (Fig. 2e) and fourfold increase in escape response time (Fig. 2f) in 72 hpf larvae.

Having confirmed the presence of disease-specific behavioural phenotypes, we next determined whether cholesterol biosynthesis and levels were indeed defective. Filipin staining of 75 µM AY9944 treated zebrafish sections, fixed at 72 hpf, was reduced compared to controls (Supplemental Figure 1c), indicative of a cholesterol biosynthetic defect, as observed in other SLOS models and patients (Xu et al. 2011; Marcos et al. 2007; Porter 2008). Despite a clear reduction in filipin staining, this change is made more difficult to observe by the quality of the fish sections.

From our data it is clear that classical cryo-sectioning is of limited value for studying lipid distribution in zebrafish embryos. This is particularly evident when attempting to compare staining intensity in brain areas of different fish that are not always perfectly matched. When considered in conjunction with the potential for artefactual changes caused by mechanical tissue manipulation (Griffiths et al. 1993; Takatori and Fujimoto 2015), it is clear that a different approach may be required. These difficulties may explain the relative absence of fluorescent lipid measurements in LSD zebrafish models. One solution may be the use of light sheet microscopy which allows for wholemount imaging of zebrafish larvae, with minimal manipulation (Icha et al. 2016). The optical transparency of the zebrafish allows detection of stained brain regions by this method with detail that would not often be possible in cryo-sections. We, therefore, tested whether light sheet microscopy of 96 hpf whole-mount zebrafish larvae could produce clearer, more detailed images of the lipid changes induced by these treatments. A particular strength of light sheet microscopy is that it allows the generation of deconvolved images from multiple planes with much greater penetration than confocal microscopy, and without the interference from incident light found in wholemount widefield fluorescence images. This allows assessment of changes in lipid levels in zebrafish organs without any loss of information caused by optical sectioning, and gives the clearest indication of the lipid burden of the whole organism, which we address in the next sections.

### Light sheet imaging of filipin

**Fig. 3:**
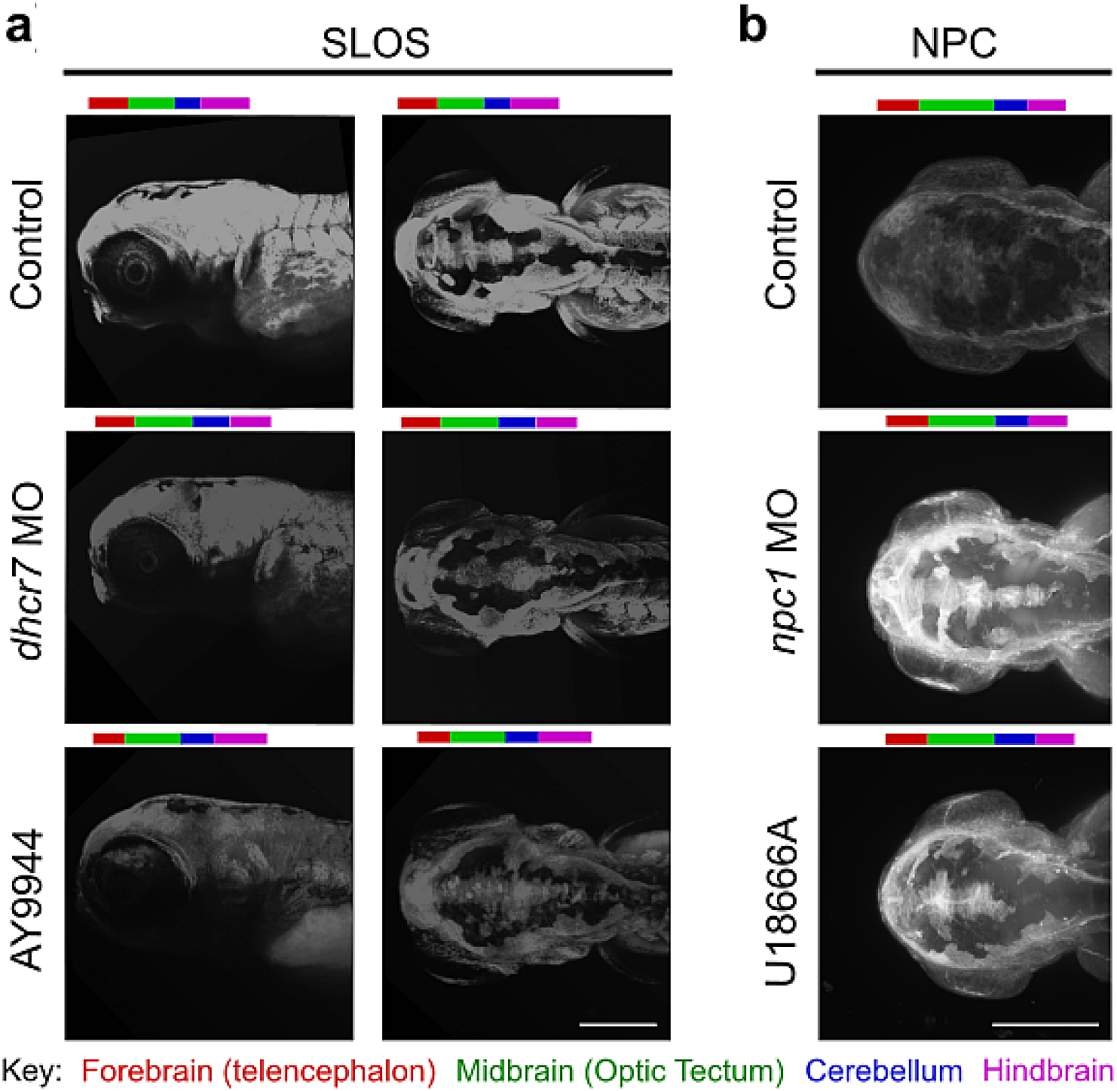
Cholesterol is reduced in SLOS model zebrafish and increased in NPC model zebrafish. Representative images of filipin staining of 96 hpf zebrafish to observe cholesterol distribution; **a** Filipin staining of SLOS zebrafish models induced using either the Dhcr7 inhibitor AY9944 (75 µM) or *dhcr7* morpholino (*dhcr7* MO) injected at the 1–2 cell stage. Images of the zebrafish head and upper body from a lateral view (left) or dorsal view (right); **b** Filipin staining of NPC zebrafish models induced using either *npc1* morpholino (*npc1* MO) injected at the 1–2 cell stage or the Npc1 inhibitor, U18666A (2 µg/ml). Images of the zebrafish head from a dorsal viewpoint. Brain areas are indicated by coloured bars above images. Scale bars = 280 µm. *N* = 3, with a minimum of ten fish per *N*

Filipin staining in the SLOS model fish confirmed a reduction in overall staining, indicating a reduced level of cholesterol, in both *dhcr7* morphant and 75 µM AY9944 treated fish compared to control (Fig. 3a), confirming the reduced cholesterol levels observed in cryosections of AY9944 treated fish (Supplemental Figure 1c). However, there is considerably more clarity in these light sheet images and considerably improved structural integrity. SLOS larvae (morphant and AY9944-treated) were imaged in lateral and dorsal orientations. The lateral orientation demonstrates that treatment with 75 μM AY9944 led to the greatest reduction in filipin staining. This is consistent with the defects in heart rate and escape response identified in Fig. 2. We do not believe that these differences are due to off-target effects of AY9944, as the difference is only in phenotype severity: no additional abnormalities were detected in AY9944 treated fish. We propose that the differences are due to potent inhibitory effects of AY9944 against Dhcr7 [IC_50_ 13 nM for human DHCR7 (Moebius et al. 1998)] and a milder effect of the morpholino. This may be due to the presence of a putative *dhcr7* paralogue, *a9ult1*, also known as si:ch1073-429i10.1, identified by the Zebrafish Mutation Project (Kettleborough et al. 2013), which is not targeted by our *dhcr7* morpholino but may be inhibited by AY9944.

In comparison, the dorsal orientation gives a clear view of staining within the brain in control fish. Cholesterol is present within the olfactory bulb, telencephalon, optic tectum, and cerebellum (see Supplemental Figure 3 for zebrafish brain regions). These brain regions can also be identified in the morpholino and AY9944 treated fish; however, staining is reduced in all, with greatly reduced filipin staining in the optic tectum. This suggests that no particular area of the brain is more affected than any other by the loss of Dhcr7 activity and that *dhcr7* is expressed across all zebrafish brain areas. This is not surprising, as cholesterol is a fundamental component of all cellular membranes, where it regulates membrane fluidity and the organisation of membrane microdomains (Lloyd-Evans and Waller-Evans 2020). A reduction in cholesterol, and presumptive accumulation of the toxic precursor, 7-DHC (Wassif et al. 1998), throughout the brain is also consistent with the pleiotropic developmental defects and global developmental delay seen in SLOS patients (Nowaczyk and Wassif 1993–2000).

Comparatively, filipin staining in NPC model fish was increased overall compared to control (Fig. 3b), indicative of cholesterol storage, and confirming the elevated staining seen in the NPC model fish sections (Supplemental Figure 1a). However, with light sheet imaging, this storage can be observed with much greater clarity and can be localised to particular brain areas. It should be noted that the level of staining in these control zebrafish appears lower than in control SLOS fish (Fig. 3a). This is a result of alterations in the imaging parameters to allow for the visualization of both reduced and increased cholesterol levels in the SLOS and NPC model zebrafish, respectively, relative to matched controls, without any excess saturation. As the control fish appear dimmer, brain regions are less easily identified. However, in the *npc1* morphant, a clear increase in staining can be seen in the olfactory bulb, telencephalon and optic tectum. The cerebellum and hindbrain, not visible in the control, are also strongly stained. While 2 µg/ml U18666A causes a less pronounced increase than the morphant, the olfactory bulb, telencephalon, cerebellum and, to an even greater degree, the optic tectum, all have strongly increased filipin staining compared to the control. Morphant fish had the brightest filipin staining, suggesting stronger loss of Npc1 function following morpholino knock down of *npc1* than with U18666A. This could be explained by inhibition of cholesterol biosynthesis or potential up-regulation of Npc1 expression in the 2 µg/ml U18666A treated fish, as has been suggested to happen in mammals (Bae and Paik 1997; Watari et al. 2000). Equally, this could reflect an impairment of U18666A transport across the chorion. As is the case with other NPC animal models, and similar to SLOS, this indicates that multiple brain regions are impaired in the NPC model zebrafish. Again, this is unsurprising, as the NPC1 protein is ubiquitously expressed in mammals (Ramirez et al. 2011). The finding of high levels of cholesterol storage in the cerebellum is another indicator that both the U18666A and *npc1* morpholino models accurately replicate NPC.

### Light sheet imaging of CtxB

Light sheet microscopy clearly demonstrated the expected changes in cholesterol levels in the SLOS and NPC model zebrafish. We, therefore, determined whether light sheet imaging could be used to study changes in the levels of ganglioside GM1, a common secondary storage molecule in LSDs, using CtxB.

**Fig. 4:**
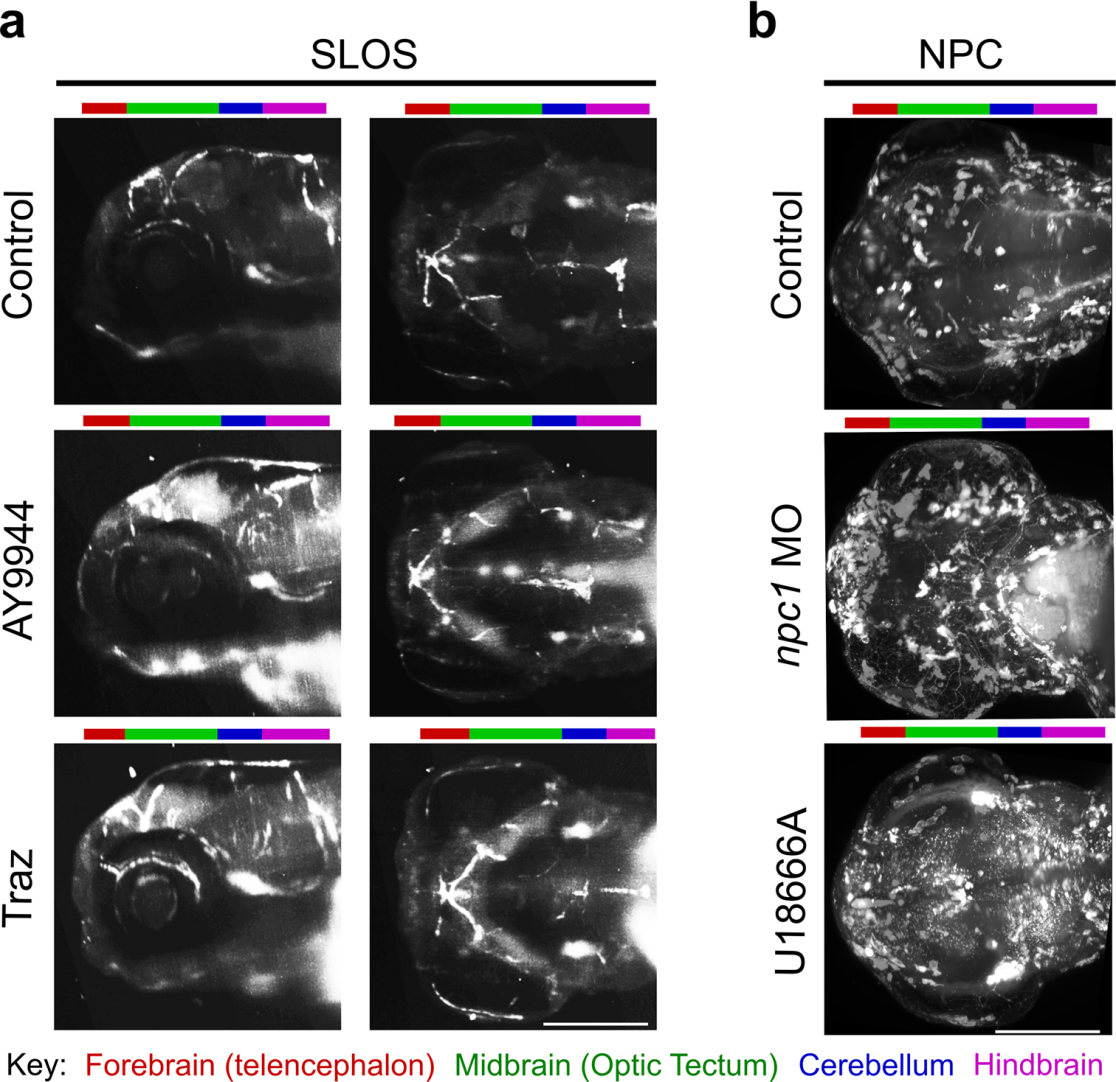
Ganglioside GM1 is increased in both SLOS and NPC zebrafish models. Representative images of cholera toxin subunit B (CtxB) staining of 96 hpf zebrafish to observe ganglioside GM1 distribution; **a** CtxB staining of SLOS zebrafish models induced using the Dhcr7 inhibitors AY9944 (75 µM) or trazodone (traz) (75 µM). Images of the zebrafish head from a lateral view (left) or dorsal view (right); **b** CtxB staining of NPC zebrafish models induced using either *npc1* morpholino (*npc1* MO) injected at the 1–2 cell stage or the Npc1 inhibitor, U18666A (2 µg/ml). Images of the zebrafish head from a dorsal viewpoint. Brain areas are indicated by coloured bars above images. Please note that the NPC images were taken using a separate Z.1 light sheet microscope that had been set up slightly differently. Scale bars = 280 µm. *N* = 3, with a minimum of ten fish per *N*

We first examined the drug-induced SLOS model zebrafish and observed an unexpected increase in CtxB staining at 96 hpf compared to controls (Fig. 4a), with the increase visibly greatest in the 75 µM AY9944 treated fish. Although this was not tested in *dhcr7* morphant zebrafish, we are confident that this increase is a real SLOS phenotype, as it is present in both drug treatments, and elevated ganglioside levels are found in the widely used AY9944 rat SLOS model (data not shown) (DeBarber et al. 2011). Most of the increases in the 75 µM AY9944 treated fish were in the brain and lower jaw regions as seen from the lateral orientation, with increases seen from the dorsal viewpoint in the olfactory bulb, optic tectum, and hindbrain, seen from both orientations. It is not surprising to see changes in ganglioside GM1 concentrations in the lower jaw of the zebrafish as facial developmental abnormalities are common in SLOS; in particular, micrognathia (small lower jaw) has been observed in more than 2/3rds of SLOS patients, indicating that this structure is particularly susceptible to loss of DHCR7 activity (Scalco et al. 2006). In the 75 µM trazodone treated fish we observed increased staining in the olfactory bulb and hindbrain, and also in the eye, which could be related to the trazodone-induced retinal area defect (Fig. 2b). The increases observed in ganglioside GM1 are likely due to a change in metabolism: reduced cholesterol levels could cause increased membrane fluidity, necessitating the insertion of additional lipid ordered domains which are ganglioside dense (Tulenko et al. 2006; Cecchi et al. 2009).

Similarly, CtxB staining was also increased, as expected, in the both the morphant and 2 µg/ml U18666A NPC model fish compared to the control, confirming the secondary storage of ganglioside GM1 in NPC fish (Fig. 4b), also observed in the NPC mouse and cat models (Vite et al. 2015; Zervas et al. 2001a). However, the pattern of increased staining in NPC models compared to controls is clearly very different to that in the SLOS models. The increased CtxB staining in the *npc1* morphant is localised around the olfactory bulb, optic tectum and in the cerebellum. Treatment with 2 µg/ml U18666A induced a more general increase in CtxB staining, similar to the global increase in filipin staining observed in these fish. The regions with the greatest accumulation of ganglioside GM1 are the olfactory bulb, optic tectum and cerebellum, the same areas that store in the morphant fish. This suggests that these areas are more severely affected by the loss of Npc1 activity, possibly because of higher levels of Npc1 expression, resulting in a higher storage burden. As with the filipin staining, it was impossible to discern this level of detail from CtxB stained cryosections, which merely indicated a general increase in ganglioside GM1 levels.

The patterns of cholesterol and ganglioside GM1 storage in the NPC model fish are not identical, with the filipin staining indicating a much more general increase in cholesterol storage throughout the fish. However, both stains indicate that the most severe storage is found in the olfactory bulb, optic tectum, and cerebellum. While this study focuses on lipid accumulation at the organ level, these staining patterns are consistent with reports in mouse and cat models of NPC, where filipin and anti-ganglioside antibodies localise both lipids to the same neurons in NPC mice (Zervas et al. 2001a; Vite et al. 2015) and in patient cells (Bergamin et al. 2013). However, the pattern of ganglioside GM1 accumulation is more spatially restricted than that of cholesterol accumulation. This suggests that the accumulation of cholesterol in NPC model fish is not entirely dependent on the accumulation of gangliosides, as has been suggested in mice (Gondré-Lewis et al. 2003).

### Conclusion

To make the most of this type of imaging it would be ideal to co-stain the fish with markers for several lipids at once, as there are many available lipid stains that work well in zebrafish and are easily detectible by light sheet microscopy, detailed in Table 1. The use of several markers would allow comparison of localisations within a single fish, from multiple angles, which could provide valuable detailed information about lipid storage in many LSDs. Another important use of light sheet microscopy would be combining mutant fish with morpholinos against sphingolipid biosynthetic genes to investigate which pathways contribute to lipid storage and are, therefore, potential drug targets. This would provide a much faster method for uncovering mechanistic information about disease pathogenesis and identifying novel drug targets, as data could be generated within 5 days using a zebrafish model, compared to the several months needed to generate and obtain the same information in mouse models.

**Table 1:** Lipid probes used in fluorescence and confocal imaging in zebrafish

Lipid	Probe	Conditions	References
Sphingolipids			
Glycosphingolipids	Periodic acid Schiff (for polysaccharides)	Fixed, HC	Kim et al. (2013)
Ganglioside GM1	Antibody	Fixed IHC	Viljetić et al. (2012)
Ganglioside GM1	Cholera toxin B	Fixed, IHC	Runft et al. (2014)
Ganglioside GM2	Antibody	Fixed, IHC	Boutry et al. (2018)
Ganglioside GM4	Antibody		Chisada et al. (2009)
Sulfatide	Toluidine blue for sulphated GSLs (also polysaccharides and nucleic acid	Fixed, HC	Kim et al. (2013)
Other lipids			
Cholesterol	Filipin	Fixed, HC	Kim et al. (2013, Louwette et al. (2013)
Neutral lipids	Oil Red O	Fixed, HC	Kadereit et al. (2008). Kim et al. (2013), Raldúa et al. (2008)

*HC *histochemistry, *IHC *immunohistochemistry, *GSLs *glycosphingolipids

The use of zebrafish models, especially in rare disease research, is dramatically increasing due to their numerous advantages over more traditional model organisms. Zebrafish often make very good LSD models, for example in CLN3 and Gaucher diseases where zebrafish models most closely recapitulate the human disease (Wager et al. 2016; Keatinge et al. 2015). We hope that the use of light sheet microscopy in zebrafish models of rare disease will allow more rapid progression of the field, yielding invaluable breakthroughs in our understanding of these diseases.

## Electronic supplementary material

Below is the link to the electronic supplementary material.

Supplementary file1 Supplemental Figure 1: Changes in cholesterol and ganglioside GM1 levels are difficult to detect in NPC and SLOS zebrafish model cryosections. Representative images of filipin and cholera toxin subunit B (CtxB) staining of 72 hpf zebrafish sections, showing the larvae head; a) Filipin staining of NPC zebrafish models induced using either npc1 morpholino (npc1 MO) injected at the 1-2 cell stage or the Npc1 inhibitor, U18666A (2 µg/ml); b) CtxB staining of NPC zebrafish models induced using either npc1 morpholino (npc1 MO) injected at the 1–2 cell stage or the Npc1 inhibitor, U18666A (2 µg/ml); c) Filipin staining of SLOS zebrafish models induced using the Dhcr7 inhibitor AY9944 (75µM). Scale bars = 280 µm. N = 3-5, with a minimum of 10 fish per N. (TIF 2145 kb)

Supplementary file2 Supplemental Figure 2: Filipin and CtxB are specific probes, and the lipids they detect are present in zebrafish larvae. A) Filipin and CtxB are specific to cholesterol and ganglioside GM1 respectively. Lipid dot blots probed with (A) filipin and (B) CtxB showing specific binding. 1 – cholesterol, 2 – sphingomyelin, 3 – ceramide, 4 – sphingosine, 5 – glucosylceramide, 6 – lyso-(bis)phosphatidic acid, 7 – phosphatidylserine, 8 – mixed gangliosides, 9 – ethanol, 10 – chloroform:methanol. N=3. B) Thin layer chromatography of 72 hpf zebrafish embryo total lipid extracts. The position of ceramide and globoside Gb4 standards were determined separately and are included here in relation to the lipids that routinely run above them (e.g. cholesterol is above ceramide, sphingomyelin is above globoside Gb4). (TIF 1559 kb)

Supplementary file3 Supplemental Figure 3: Diagrams representing zebrafish brain anatomy from dorsal and lateral viewpoint. A) Schematic diagram of zebrafish brain areas in lateral and dorsal orientations. B) Image of filipin stained control fish from Figure 3a, with brain regions pseudocoloured. (EPS 1383 kb)
