# Supplementary figures and images for: Visualisation of cholesterol and ganglioside GM1 in zebrafish models of Niemann–Pick type C disease and Smith–Lemli–Opitz syndrome using light sheet microscopy
Source: Histochem Cell Biol. 2020 Oct 20;154(5):565–78. doi: 10.1007/s00418-020-01925-2 (PMC7609433; doi:10.1007/s00418-020-01925-2)

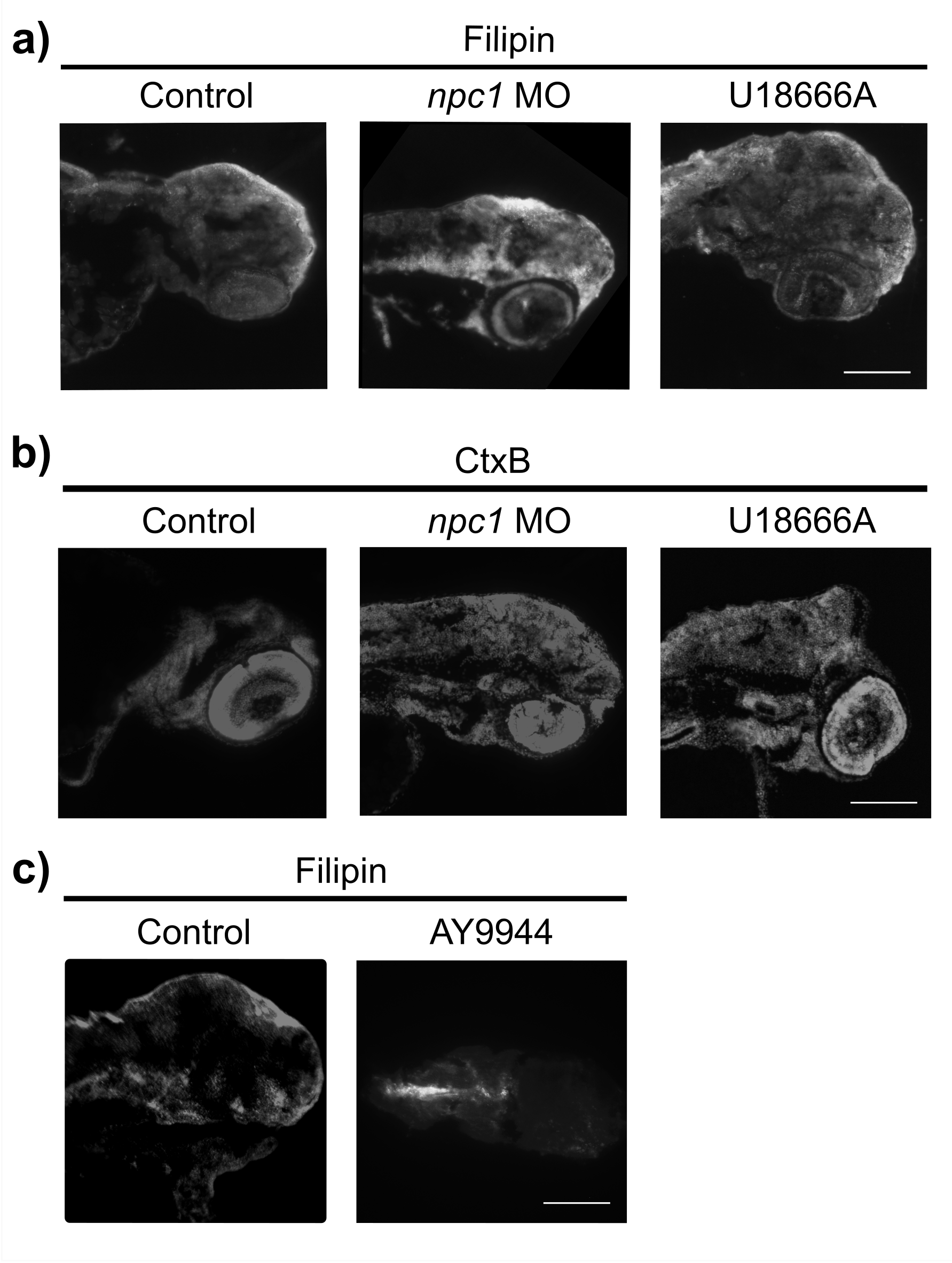

Supplement: Supplementary file 1 — Supplementary file1 Supplemental Figure 1: Changes in cholesterol and ganglioside GM1 levels are difficult to detect in NPC and SLOS zebrafish model cryosections. Representative images of filipin and cholera toxin subunit B (CtxB) staining of 72 hpf zebrafish sections, showing the larvae head; a) Filipin staining of NPC zebrafish models induced using either npc1 morpholino (npc1 MO) injected at the 1-2 cell stage or the Npc1 inhibitor, U18666A (2 µg/ml); b) CtxB staining of NPC zebrafish models induced using either npc1 morpholino (npc1 MO) injected at the 1–2 cell stage or the Npc1 inhibitor, U18666A (2 µg/ml); c) Filipin staining of SLOS zebrafish models induced using the Dhcr7 inhibitor AY9944 (75µM). Scale bars = 280 µm. N = 3-5, with a minimum of 10 fish per N. (TIF 2145 kb) [file 418_2020_1925_MOESM1_ESM.tif]

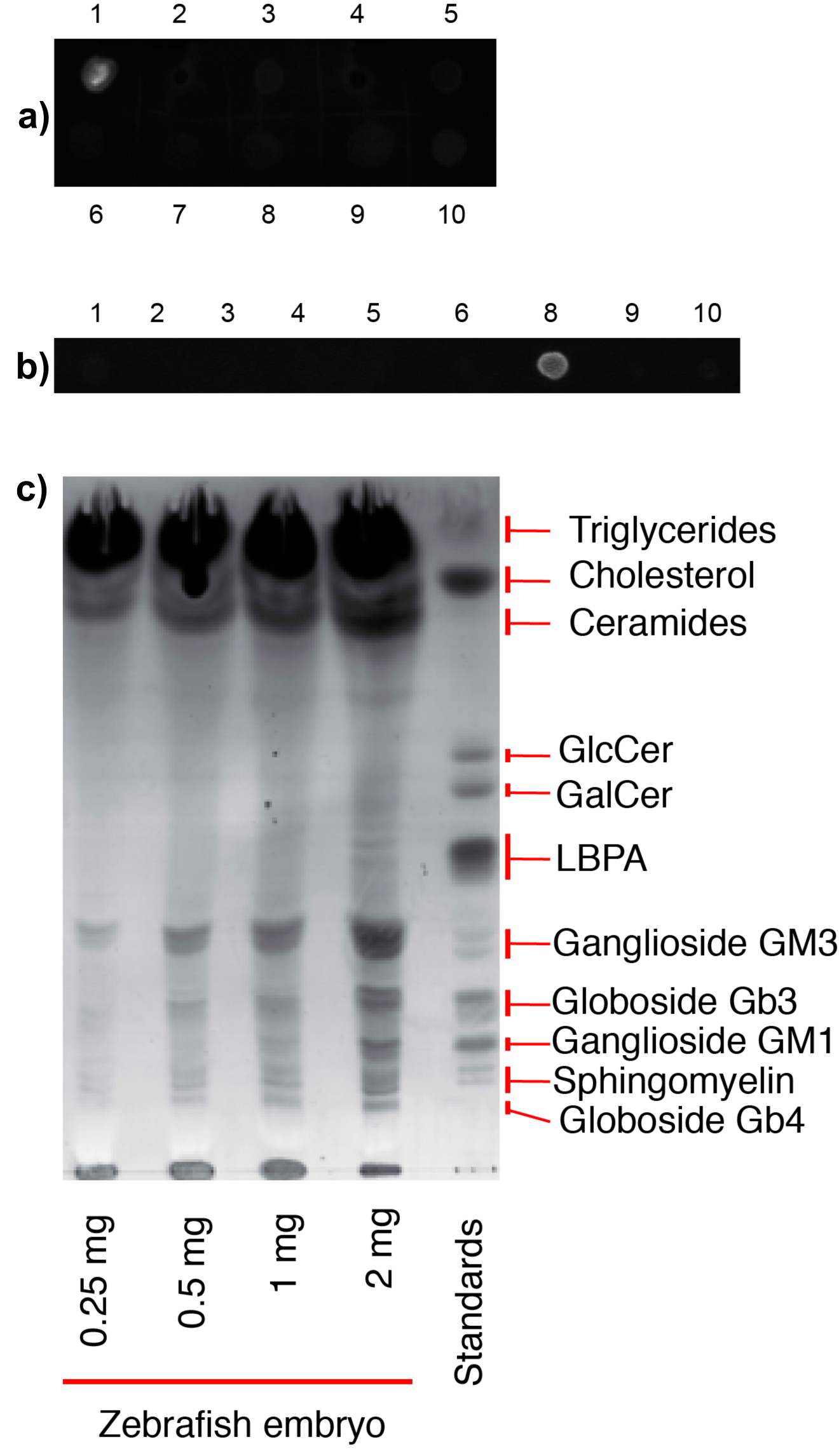

Supplement: Supplementary file 2 — Supplementary file2 Supplemental Figure 2: Filipin and CtxB are specific probes, and the lipids they detect are present in zebrafish larvae. A) Filipin and CtxB are specific to cholesterol and ganglioside GM1 respectively. Lipid dot blots probed with (A) filipin and (B) CtxB showing specific binding. 1 – cholesterol, 2 – sphingomyelin, 3 – ceramide, 4 – sphingosine, 5 – glucosylceramide, 6 – lyso-(bis)phosphatidic acid, 7 – phosphatidylserine, 8 – mixed gangliosides, 9 – ethanol, 10 – chloroform:methanol. N=3. B) Thin layer chromatography of 72 hpf zebrafish embryo total lipid extracts. The position of ceramide and globoside Gb4 standards were determined separately and are included here in relation to the lipids that routinely run above them (e.g. cholesterol is above ceramide, sphingomyelin is above globoside Gb4). (TIF 1559 kb) [file 418_2020_1925_MOESM2_ESM.tif]
